# Coordination between scientific and technological innovation and the high-quality development of Baijiu industry: The coupling and decoupling perspective

**DOI:** 10.1371/journal.pone.0301589

**Published:** 2024-05-07

**Authors:** Zhixia Wu, Xiazhong Zheng, Yijun Chen, Shan Huang, Chenfei Duan, Wenli Hu

**Affiliations:** 1 College of Management, Sichuan University of Science & Engineering, Zigong, 643000, China; 2 College of Hydraulic & Environmental Engineering, China Three Gorges University, Yichang, 443002, China; 3 College of Economics & Management, China Three Gorges University, Yichang 443002, China; 4 College of Architecture and Urban-Rural Planning, Sichuan Agricultural University, Dujiangyan, 611830, China; Inner Mongolia University, CHINA

## Abstract

The Baijiu industry is a significant contributor to both the food industry and the light industry. Its high tax characteristics effectively promote the sustainable development of the regional economy. First, the evaluation index system of scientific and technological innovation (STI) and high-quality development of Baijiu industry (HQDBI) were constructed. The entropy-improved CRITIC method was used to measure the weights. Second, the coordination relationship and evolution trend of STI and HQDBI were explored using the coupling coordination model and the Tapio decoupling model. Then, the transfer law and key influencing factors were further investigated using the Markov chain and grey correlation, respectively. The main contribution is the dynamic evolution of the coupling and decoupling relationships from the perspective of multiple Baijiu provinces, and deeply depicts the coordination relationship and evolutionary trends of STI and HQDBI. The results show that: the spatial distribution of the coupling coordination degree shows high values in the east-west and low values in the north-south characteristics. In 2021, a pattern of coordinated development in Baijiu provinces has emerged along the Yangtze River basin. The decoupling state is mainly strong decoupling, but it remains poor in Shanxi. The coordination process is unstable and difficult to achieve leapfrog development. Coordination, sustainability and innovation environment have a greater impact on the coordination of subsystems.

## 1 Introduction

Chinese Baijiu is one of the world’s top six distilled spirits, alongside Brandy, Whisky, Vodka, Rum and Gin [1,2]. The Baijiu industry is an important part of the food industry [3–5], and is known for its high tax revenue characteristics, which effectively enhance the resilience of the regional economy [2,6]. Scientific and technological innovation (STI) is crucial for modern economic growth [7,8], providing technological support and a fundamental guarantee for improving social productivity [9–11]. During the new development period, STI plays a crucial role in promoting sustainable industrial growth [12,13]. STI can contribute to addressing environmental and socio-economic issues [14,15], as well as enhancing the high-quality development of Baijiu industry (HQDBI) [16,17]. In turn, implementing the new development concept and promoting the HQDBI can further strengthen the effect of STI [18] and enhance the core competitiveness of Baijiu industry [19]. It can be seen that STI and HQDBI show interrelated and mutually reinforcing interlocking influences [20], there may be a coupling coordination relationship [12]. However, while the Baijiu industry can bring significant economic benefits to local areas, there are also challenges in its development, such as overcapacity and increasing industry differentiation [2]. As an important embodiment of the light industry, the coordination evaluation of Baijiu industry is crucial in promoting HQDBI and thus promoting sustainable socio-economic development [21].

Baijiu industry is currently undergoing a crucial phase of high-quality economic development and industrial adjustment. Internally, it faces the problem of eliminating outdated production capacity and the market’s supply-demand imbalance [20]. Externally, it faces the problem of the crowding-out effect of economic uncertainty. Second, with the upgrading of consumption, the effective supply is facing challenges, and competition within Baijiu industry is expected to intensify in the future. It is evident that HQDBI is an environmentally friendly development mode characterized by stable growth, structural adjustment and improved efficiency. It is difficult to achieve only by relying on traditional brewing methods. The HQDBI should rely on STI [22]. To achieve this goal, many Baijiu enterprises are currently accelerating the improvement of their STI capacity and level. The focus is on using STI to promote the optimization of traditional Baijiu technology and advance Baijiu achievements. This is exactly one important way to ensure the sustainable development of Baijiu industry.

Coupling and decoupling are important tools for analyzing the coordination of multiple systems [23–25]. They are now widely used in several industries or fields of sustainable development [26–29], including energy [30,31], environment [32,33] and socio-economy [34,35]. For example, Wang (2023) [12] investigated the coupling mechanism and evolutionary characteristics of STI and the ecological environment [36]. As for industry, the development of STI will increase industrial production and energy consumption [37,38]. Cheng (2022) [38] took industrial enterprises as an example to explore the coordination relationship between STI and eco-efficiency. Liang (2020) [9] analyzed the mechanism and law of the synergistic evolution between STI and regional economy, using the high-tech industry as an example. Scholars have also evaluated the coordination of STI and the economic growth subsystem [39] or explored the decoupling effect [35]. In addition, existing studies indicated that STI is a new engine for the high-quality development of Baijiu enterprises [16,17], providing a scientific-technical basis and cutting-edge tools for intelligent Baijiu brewing [20]. For example, Guo(2023) [40] noted that STI can enhance enterprises’ market share, the information management level [41], and innovation in brewing microbiology-related research [42], such as cellar mud replication [43], brewing environment microbiological replication, etc. [20,44–46].

In summary, many scholars have shown interest in researching the coordination of STI in the fields of ecological environment or economic development. However, research is mostly limited to qualitative analysis and path-type improvement. And limited to a single region, there is a lack of quantitative comparative analysis of multiple regions. In China’s major Baijiu producing regions, whether there is a coordination relationship between HQDBI and STI has not yet been clarified. Moreover, while the coupling theory can assess the coordination level of multiple systems, existing research tends to analyze decoupling and coupling separately. However, decoupling and coupling are interdependent. Based on these two perspectives, it is more beneficial to comprehensively analyze the coordination between STI and HQDBI.

Therefore, in order to further explore the mutual influence relationship between STI and HQDBI, this study analyzed the major Baijiu provinces (Guizhou, Sichuan, Jiangsu, Anhui and Shanxi) using the index systems of HQDBI and STI. First, based on the panel data from 2011 to 2021, the entropy-improved CRITIC method was used to assign weights for comprehensive index analysis. Second, the coordination relationship and evolution trend were analyzed using the coupling coordination model and the Tapio decoupling model. Then the Markov chain was used to represent the transfer law of the coupling coordination degree. The grey correlation model was used to clarify the key factors that have an impact on each indicator. This study aims to provide some references for the synergy between STI and HQDBI.

The main innovations: (1) Existing studies do not systematically consider these factors affecting HQDBI. This study identified factors that reflect the resource input and production output of Baijiu industry, including innovation input and industrial coordination. (2) The weight was calculated using the entropy-improved CRITIC method. This method objectively reflects the degree of dispersion, conflict, and contrast strength of the data, thereby reducing the adverse effects of subjective judgment. (3) Previous studies have neglected the interaction and evolution patterns between STI and HQDBI. However, this study analyzed them from the perspective of coupling and decoupling.

## 2 Materials and methods

### 2.1 Study area

**Fig 1:**
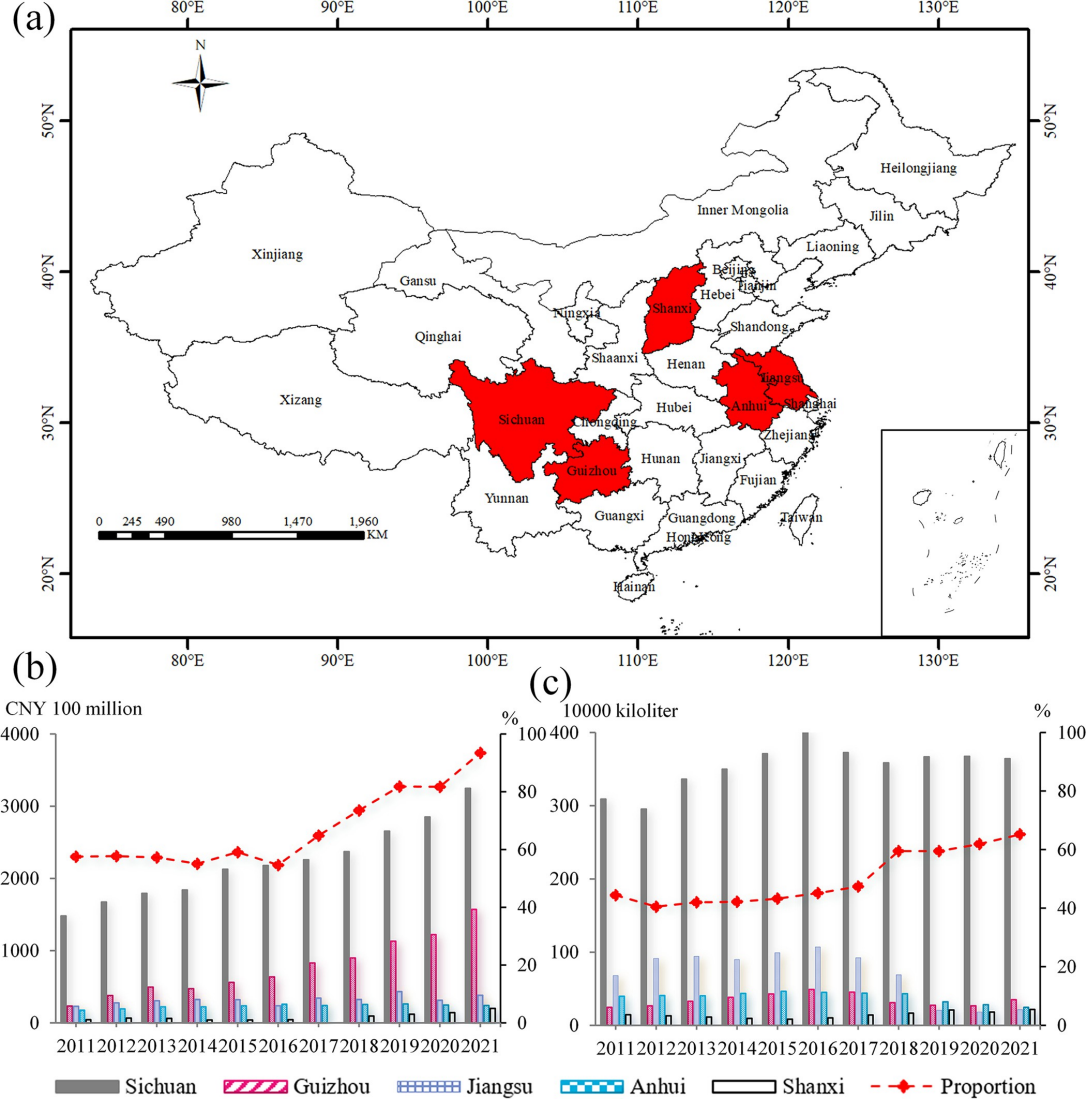
Study area. (a) Geographical distribution of the study area; (b) Operating revenue of the Baijiu industry; (c) Baijiu production.

The study area (Fig 1A) comprises five provinces that produce Baijiu. (1) Sichuan Province (97°21′-108°12′ E, 26°03′-34°19′ N) is a gold Baijiu producing region in China. The region is home to famous Baijiu brands such as Wuliangye, Langjiu and Luzhou Laojiao [47,48]. From 2011 to 2021, Sichuan Baijiu accounted for 41.39% of China’s average operating revenue (Fig 1B). (2) Guizhou Province (103°36′-109°35′ E, 24°37′-29°13′ N) is a world-renowned core production area for saucy liquor, with Baijiu brands such as Guizhou Moutai and Xi liquor. In recent years, Guizhou’s Baijiu industry profits have increased in proportion to the country’s share, from 22.43% in 2011 to 52.29% in 2021, with less than 5% of production generating half of the industry’s profits. (3) Jiangsu Province (116°18′-121°57′ E, 30°45′-35°20′ N) with a number of famous Baijiu production areas, such as Suqian City, Nanjing City, with Yanghe Shares, Jinshiyuan and other famous Baijiu listed companies. (4) Anhui Province (114°54′-119°37′ E, 29°41′-34°38′ N) has well-known Baijiu listed companies such as Yingjiagong and Golden Seed Liquor. The region has rich brewing resources and a long Baijiu culture. (5) Shanxi Province (110°14′-114°33′ E, 34°34′-40°44′N), which produces Fenjiu, one of the ten most famous Baijiu in China, is an important production area for clear-flavored Baijiu [36]. For example, from 2018 to 2021, the average operating revenue of the Baijiu industry in the five provinces accounts for more than 80% of the national revenue (Fig 1B), and Baijiu production accounts for more than 60% of China (Fig 1C).

The base map outline was obtained by using ArcGIS 10.2 based on the Service Center of Standard Map (http://bzdt.ch.mnr.gov.cn/) and the permission number is GS (2016) 2923.

### 2.2 Method

#### 2.2.1 The entropy-improved CRITIC method

Weighting is a critical step in comprehensive evaluation and subsequent measurement analysis. Objective weighting methods commonly used in existing research include the entropy method, CRITIC method and coefficient of variation method. Among these methods, the CRITIC method is a relatively effective objective weighting method. CRITIC can effectively consider the correlation and contrast between indicators, but it ignores the degree of dispersion between indicators. And the entropy method can effectively reflect the degree of discrete data. When combined with the two assignment methods, it can more comprehensively reflect objective attributes, such as the degree of discreteness and contrast intensity [49,50]. This compensates for the shortcomings of each method.

(1) CRITIC method

The main steps of the CRITIC method are as follows.

First, the dimensionless processing of data.

$$Positive\;indicator:x_{ij}^{'}=\left(x_{ij}-x_{\min}\right)/(x_{\max}-x_{\min})$$ (1)

$$Negative\;indicator:x_{ij}^{'}=\left(x_{\max}-x_{ij}\right)/(x_{\max}-x_{\min})$$ (2)

*x*_*ij*_ is the raw data for the *i*th indicator in year *i*. *x*_max_ and *x*_min_ are the maximum and minimum values of the indicator, respectively.

Next, the standard deviation of the *j*th indicator is calculated.

$$\sigma_{j}=\sqrt{\frac{\sum_{i=1}^{n}{\left(x_{ij}^{'}-\sum_{i=1}^{n}x_{ij}^{'}/n\right)}^{2}}{n}}$$ (3)

*σ*_*j*_ is the size of the gap for the indicator importance index.

Third, quantitative conflict indicators are calculated.

$$y_{j}=\sum_{i=1}^{n}\left(1-r_{ij}\right)$$ (4)

*y*_*j*_ is the conflict of the *j*th indicator with other indicators. *r*_*ij*_ is the correlation coefficient between indicators *i* and *j*.

Fourth, the weights of the CRITIC method are calculated.


$$\omega_{j(\mathrm{CRITIC})}=\sigma_{j}y_{j}/\sum_{j=1}^{m}\sigma_{j}y_{j}$$ (5)

(2) Entropy method

Entropy method weights are calculated based on the dimensionless results.

$$e_{j}=-(\frac{1}{\ln m})\sum_{i=1}^{m}(x_{ij}^{'}/\sum_{i=1}^{m}x_{ij}^{'})\times \ln(x_{ij}^{'}/\sum_{i=1}^{m}x_{ij}^{'})$$ (6)

$$\omega_{j(\mathrm{Entropy})}=\left(1-e_{j}\right)/\sum_{j=1}^{m}(1-e_{j})$$ (7)

*e*_*j*_ is the entropy value of the *j*th indicator.

(3) Improved CRITIC method

First, it is evident from the above steps that the correlation coefficient *r*_*ij*_ can be either positive or negative. However, *r*_*ij*_ with the same absolute value indicates equivalent indicator correlations. Therefore, it is more appropriate to use 1−|*r*_*ij*_| instead of 1−*r*_*ij*_ when reflecting the contrast between indicators. Second, the inclusion of entropy can fully account for the conflicting, contrasting and dispersion of indicators. In summary, based on the results of previous research [49,50], the improved formula is as follows:

$$\omega_{j}=(e_{j}+\sigma_{j})\sum_{i=1}^{n}\left(1-|r_{ij}|\right)/\sum_{j=1}^{m}\left(e_{j}+\sigma_{j}\right)\sum_{i=1}^{n}\left(1-|r_{ij}|\right)$$ (8)

The comprehensive index of STI and HQDBI is measured as follows.


$$S_{i}^{t}=\sum_{j=1}^{m}\omega_{j}x_{ij}$$ (9)

$S_{i}^{t}$ is the comprehensive index for the *i*th subject in year *t*.

#### 2.2.2 Coupling coordination model

From a systems theory perspective, STI and HQDBI form a complex system that interacts and influences each other [38,51,52]. The coupling model can measure the degree of interaction between systems, but it does not reflect the specific effects of the interacting forces nor indicate the level of coordinated development. The coupling coordination model can better compensate for this shortcoming, and describe the degree of interaction and coordination between the elements within the system [51]. Therefore, this study used the coupling coordination model to analyze the overall coordination degree of STI and HQDBI, which helps to gain a deeper understanding of the coordination mechanisms.


$$C=\sqrt{\frac{U_{1}U_{2}}{\{\left(U_{1}+U_{2}\right)/{2\}}^{2}}}$$ (10)

$$D=\sqrt{CT}$$ (11)

$$T=\alpha U_{1}+\beta U_{2}$$ (12)

*U*_*1*_ and *U*_*2*_ are the comprehensive evaluation functions of STI and HQDBI systems. *C* is the coupling degree. *D* is the coupling coordination degree. *T* is the comprehensive index of the STI and HQDBI. *α* and *β* are the set weights, often set to 0.5 [53,54]. Coupling coordination levels are divided into five main categories and 15 subcategories [55,56] (Table 1).

**Table 1:** Coupling coordination levels.

Categories	Classification criteria	Subcategories	Numerical relationship of the comprehensive index
Severe disorder	0≤*D*<0.4	Severe disorder—STI is hindered	*U*_2_−*U*_1_>0.1
		Severe disorder—HQDBI is hindered	*U*_1_−*U*_2_>0.1
		Severe disorder	*|U*_1_−*U*_2_|<0.1
Primary disorder	0.4≤*D*<0.5	Primary disorder—STI is hindered	*U*_2_−*U*_1_>0.1
		Primary disorder—HQDBI is hindered	*U*_1_−*U*_2_>0.1
		Primary disorder	*|U*_1_−*U*_2_|≤0.1
Primary coordination	0.5≤*D*<0.6	Primary coordination—STI lags behind	*U*_2_−*U*_1_>0.1
		Primary coordination—HQDBI lags behind	*U*_1_−*U*_2_>0.1
		Primary coordination	|*U*_1_−*U*_2_|≤0.1
Well coordination	0.6≤*D*<0.8	Well coordination—STI lags behind	*U*_2_−*U*_1_>0.1
		Well coordination—HQDBI lags behind	*U*_1_−*U*_2_>0.1
		Well coordination	|*U*_1_−*U*_2_|≤0.1
Wonderful coordination	0.8≤*D*<1	Wonderful coordination—STI lags behind	*U*_2_−*U*_1_>0.1
		Wonderful coordination -HQDBI lags behind	*U*_1_−*U*_2_>0.1
		Wonderful coordination	|*U*_1_−*U*_2_|≤0.1

#### 2.2.3 Tapio decoupling model

Decoupling is the separation of the system [57–60]. The Tapio decoupling model is characterized by accuracy and independence from statistical measures [61]. Its core involves subdividing decoupling states by introducing the decoupling elasticity coefficient, which is an effective tool for decoupling analysis [60]. The decoupling elasticity coefficient is constructed using the Tapio decoupling model. This study analyzed the coordination between STI and HQDBI by combining the Tapio decoupling model with the coupling coordination model.


$$DE=\frac{\Delta TI}{\Delta HQ}=\frac{\left(TI_{t+k}-TI_{t}\right)/TI_{t}}{\left(HQ_{t+k}-HQ_{t}\right)/HQ_{t}}$$ (13)

Where *TI*_*t*_ and *TI*_*t+k*_ are the STI index for the years *t*, *t+k*, and *HQ*_*t*_ and *HQ*_*t+k*_ are the index of HQDBI for the years *t*, *t+k*. *DE* is the decoupling elasticity index. The decoupling state is divided into eight categories [62], as shown in Table 2.

**Table 2:** Classification of decoupling state.

Decoupling state		Δ*TI*	Δ*HQ*	*DE*
Decoupling	Strong decoupling (SD)	-	+	*DE*<0
	Weak decoupling (WD)	+	+	0≤*DE*<0.8
	Recession decoupling (RD)	-	-	*DE*≥1.2
Connection	Growth connection (GC)	+	+	0.8≤*DE*<1.2
	Recession connection (RC)	-	-	0.8≤*DE*<1.2
Negative decoupling	Weak negative decoupling (WN)	-	-	0≤*DE*<0.8
	Expansion negative decoupling (EN)	+	+	*DE*≥1.2
	Strong negative decoupling (SN)	+	-	*DE*<0

#### 2.2.4 Coordinating influence model

To measure the influence of the subsystems on the coordination degree, a coordinating influence model was constructed [53]. This is a valuable method for analyzing the relationship between subsystems and coupling coordination.


$$CI=W_{x}(D_{x}-D_{y})$$ (14)

*D* is the system of STI (HQDBI), *D*_*x*_ is the coupling coordination degree of STI (HQDBI) and HQDBI (STI) subsystems. *D*_*y*_ is the coupling coordination degree of the two systems.

#### 2.2.5 Markov chain

The Markov chain method is a significant tool for predicting the state of events and their developmental trends. It can also analyze the evolution law of coupling coordination. The coupling coordination level is divided into *N* states and a matrix *P* consisting of N*N state transfer probability is constructed.

$$P=(\begin{array}{ccc} m_{11} & \ldots & m_{1k}\\ \vdots & \ddots & \vdots \\ m_{k1} & \cdots & m_{kk} \end{array})$$ (15)

$$m_{ij}=\frac{n_{ij}}{n_{i}}$$ (16)

*n*_*ij*_ is the total number of regions transferred from type *i* at time *t* to type *j* at time *t+1*. *n*_*i*_ is the total number of regions of type *i* at moment *t*.

#### 2.2.6 Grey correlation

The grey correlation method can effectively analyze the correlation between coupling coordination degree and the indicators [63]. It reflects relationships and trends between samples, making it a valuable analytical method.


$$\xi_{i}=\frac{\min_{i}\min_{k}|X_{0}(k)-X_{i}(k)|+\rho \max_{i}\max_{k}|X_{0}(k)-X_{i}(k)|}{\left|X_{0}(k)-X_{i}(k)|+\rho \max_{i}\max_{k}|X_{0}(k)-X_{i}(k)\right|}$$ (17)

$$\gamma_{ij}=\frac{1}{n}\sum_{k}^{n}\xi (k)$$ (18)

*X*_0_(*k*) is the reference sequence, $X_{0}(k)=\{x_{0}(1),x_{0}(2),\cdots ,x_{0}(k)\}$. *X*_*i*_(*k*) is the comparison sequence, $X_{i}(k)=\{x_{i}(1),x_{i}(2),\cdots ,x_{i}(k))$. $\underset{i}{\min}\underset{i}{\min}|x_{0}(k)-x_{i}(k)|$ is the bipolar minimum difference. $\underset{i}{\max}\underset{i}{\max}|x_{0}(k)-x_{i}(k)|$ is the bipolar maximum difference. *ξ*_*i*_ is the correlation coefficient.*ρ* is the discrimination coefficient, ranging from 0 to 1, and is usually equal to 0.5. *γ*_*ij*_ is the degree of correlation.

### 2.3 Data sources and processing

The data used in this study was obtained from various sources, including the China Science and Technology Statistical Yearbook (2012–2022), the China High-Tech Industry Statistical Yearbook, the National Technology Market Statistical Annual Report, and the Statistical Yearbooks of each province in previous years. Additionally, the data was obtained from the annual reports of listed Baijiu companies from 2012 to 2022 and the China Wine Industry Association. Some of the missing values were interpolated. Standardization data processing was used to eliminate the influence of dimensionality.

## 3 Selection of indicators

The evaluation index system (Table 3) of HQDBI was constructed based on the five development concepts [64], the connotation of high-quality development [6,65], and considering the applicability and availability of data [24]. (1) To directly reflect the impact of innovation output on HQDBI, I3 and I4 were selected as innovation indicators. Due to the specific nature of Baijiu industry and the Matthew effect [6], HQDBI cannot be separated from the resource investment of the regional liquor enterprises. Although innovation input alone may not fully reflect the impact of real industry input, we also considered R&D expenses (I_1_) and R&D personnel (I_2_). (2) In terms of coordination, regional coordination, industry coordination and people’s livelihood coordination reflect the balance and interaction of regional Baijiu industry. Based on existing studies, regional coordination is reflected in two aspects, C1 [66] and C2. Industrial coordination is complemented by C4 and C5 indicators, which are based on the existing indicator C3 [6]. Scholars have suggested that C6 and C7 are the important indicators of livelihood coordination [67]. (3) For sustainability, S1 [68] and S2 [69] can reflect the foundation of regional development and the level of human capital, which can contribute to the stable growth of Baijiu industry. The growth of Baijiu industry cannot be separated from the stable development of enterprise scale and production [70], so S3, S4 and S5 are selected. In addition, environmental protection expenditures reflect green development. (4) For openness and sharing, O1 and O2 reflect the foreign trade of the Baijiu industry [66]. O3 reflects both foreign and domestic revenue of Baijiu. The tertiary indicator’s attributes are determined based on the previous division.

**Table 3:** Evaluation index system of HQDBI.

Primary indicators	Secondary indicators	Tertiary indicators	Attribute
Innovation (I)	Innovation input	R&D expenses of Baijiu enterprises (I_1_)	+
		R&D personnel of Baijiu enterprises (I_2_)	+
	Innovation output	Contribution rate of Baijiu industry (I_3_)	+
		Labour productivity of Baijiu industry (I_4_)	+
Coordination (C)	Regional coordination	Income ratio of urban-rural residents (C_1_)	-
		Ratio of average annual Baijiu consumption of urban-rural residents (C_2_)	-
	Industry coordination	Market share of Baijiu (C_3_)	+
		Output per unit area of Baijiu industry (C_4_)	+
		Advanced structure of Baijiu industry (C_5_)	+
	People’s livelihood coordination	Baijiu consumption price index (C_6_)	-
		Annual per capita consumption expenditure of Baijiu (C_7_)	+
Sustainability (S)	Stable development	Level of regional economic development (S_1_)	+
		Employment stability (S_2_)	+
	Stable growth	Profit of Baijiu industry (S_3_)	+
		Number of above-scale Baijiu enterprises (S_4_)	+
		Ratio of annual Baijiu production to China’s annual Baijiu production (S_5_)	+
	Green development	Environmental protection expenditure (S_6_)	+
Openness and sharing (O)	Foreign trade	Foreign trade dependence (O_1_)	+
		Foreign capital dependence (O_2_)	+
	Openness to Win-Win	Proportion of Baijiu export revenue (O_3_)	+

Note: (1) C_3_ is the regional Baijiu industry revenue/revenue of China’s Baijiu industry revenue. C_5_ is the output value of Baijiu industry/total industry output value above scale. S_2_ is the number of employees of Baijiu enterprise /regional employees. O_3_ is the export business income of Baijiu products for Baijiu enterprise/domestic operating income. (2) Above-scale Baijiu enterprises: with an annual output of over 10,000 tons and fixed assets exceeding 100 million.

Based on STI-related research [9,12,38], an indicator system was constructed in three dimensions: innovation input, innovation output and innovation environment, as shown in Table 4. (1) Innovation inputs consist of financial and human inputs. Financial inputs include multiple expenditures, and previous studies have concluded that II1, II2 and II3 are crucial for the high-quality development of the industry [71]. STI cannot be separated from talents, and talents have a strong subjective initiative. Therefore, we choose technical talents who are truly engaged in scientific research and committed to the development of new products, i.e., II4 and II5 to examine human resource input [9]. (2) The role of STI relies on the transformation of results. According to the research topic of this study, we chose the indicators of the results transformation category that appear more frequently in existing studies, namely IO1~IO5 as direct output [38,71]. IO6 and IO7 as the results of the high-tech industry [70]. (3) IE1~IE4 were selected to measure the level of innovation environment, inspired by scholars [12].

**Table 4:** Evaluation index system of STI.

Primary indicators	Secondary indicators	Tertiary indicators	Attribute
Innovation input (II)	Financial input	Internal expenditure on R&D funds RD (II_1_)	+
		Expenditure on science and technology (II_2_)	+
		Education expenditure (II_3_)	+
	Manpower input	R&D personnel (II_4_)	+
		Number of employees in high and new technology (II_5_)	+
Innovation output (IO)	Direct output	Number of patents granted (IO_1_)	+
		Number of scientific papers (IO_2_)	+
		Technology market transaction volume (IO_3_)	+
		Number of scientific and technical publications (IO_4_)	+
		Number of signed technical contracts (IO_5_)	+
	Outputs of high-technology industry results	Sales revenue of new products in high-tech industries (IO_6_)	+
		Total profit of high-tech industries (IO_7_)	+
Innovation environment (IE)	Manpower foundation	Number of students in general higher education (IE_1_)	+
		Number of full-time teachers in general higher education (IE_2_)	+
	Institutional foundation	Number of scientific research and technology development institutions (IE_3_)	+
		Number of high-tech industry enterprises (IE_4_)	+
		Number of higher education institutions (IE_5_)	+

## 4 Results

### 4.1 The comprehensive index evaluation results of STI and HQDBI

From 2011 to 2021, the overall development level of STI in five provinces shows a fluctuating upward trend. However, there are significant differences between regions (Table 5). Jiangsu stands out with its exceptional advantages in the high-tech industry, concentrated innovation resources, and a leading comprehensive index of STI. Sichuan has a well-developed foundation of STI, but the comprehensive index shows a fluctuating downward trend. This may be due to the fact that Sichuan, as a large province in southwest China, has a better foundation for STI and has achieved remarkable results in the conversion of STI. However, the lack of innovation carriers and innovative research and development platforms has hindered the further improvement of STI environment [71]. From 2011 to 2018, Anhui’s STI has fluctuated but has shown solid improvement since 2019. The comprehensive index is below 0.1 in Guizhou and Shanxi, indicating a low overall level. This may be due to the influence of the siphoning effect. The detailed results can be found in the S1 File.

**Table 5:** Comprehensive index of STI and HQDBI in five provinces, 2011–2021.

Year	Comprehensive index of STI					Comprehensive index of HQDBI				
	Sichuan	Guizhou	Jiangsu	Anhui	Shanxi	Sichuan	Guizhou	Jiangsu	Anhui	Shanxi
2011	0.528	0.002	0.689	0.207	0.050	0.662	0.357	0.462	0.186	0.275
2012	0.545	0.001	0.668	0.176	0.045	0.592	0.439	0.499	0.194	0.240
2013	0.550	0.004	0.660	0.168	0.041	0.592	0.461	0.521	0.274	0.173
2014	0.555	0.016	0.669	0.174	0.032	0.589	0.412	0.583	0.296	0.087
2015	0.553	0.023	0.677	0.180	0.029	0.633	0.440	0.487	0.265	0.188
2016	0.538	0.032	0.687	0.205	0.023	0.504	0.512	0.456	0.274	0.214
2017	0.542	0.030	0.676	0.189	0.020	0.573	0.531	0.462	0.269	0.194
2018	0.552	0.027	0.660	0.173	0.014	0.609	0.469	0.396	0.310	0.221
2019	0.507	0.030	0.711	0.267	0.018	0.561	0.475	0.450	0.353	0.205
2020	0.493	0.029	0.719	0.306	0.017	0.576	0.447	0.432	0.315	0.255
2021	0.476	0.020	0.733	0.315	0.014	0.623	0.480	0.467	0.320	0.190
Average	0.531	0.020	0.686	0.214	0.027	0.592	0.457	0.474	0.278	0.204

The overall situation of HQDBI is better than that of STI (Table 5) in five provinces. Except for Guizhou’s short-lived lead over Sichuan in 2016, Sichuan has been far ahead of the other regions, with strong stability in HQDBI. The main reasons for the growth of the Baijiu industry in Sichuan are its solid foundation, the strong development of listed companies and the prominent advantages of famous brands. Anhui’s Baijiu industry has experienced rapid growth in recent years, with its comprehensive index rising from 0.186 (2011) to 0.320 (2021), thanks to its four listed Baijiu companies.

### 4.2 Coupling coordination analysis of STI and HQDBI

(1) Spatio-temporal characteristics

As can be seen from Table 6 (or S2 File), the five provinces do not fully achieve wonderful coordination from 2011 to 2021, and the overall coordination level is low. The coupling coordination degree between Guizhou and Anhui shows a fluctuating upward trend, with Anhui achieving a higher level of coordination. The overall coupling coordination level between Sichuan and Jiangsu ranges from 0.722 to 0.790. This indicates a relatively stable state of coordination without any disorder stage, but with very small changes. It is worth noting that in some years, the level of coupling coordination between the two provinces even declined. This may be influenced by the Chinese government’s restrictions on Baijiu consumption. Shanxi has the largest decrease in coupling coordination degree, with a reduction of 33.8%. The coordination degree reached its minimum value of 0.227 in 2021.

**Table 6:** The coupling coordination degree between STI and HQDBI, 2011–2021.

Province	2011	2012	2013	2014	2015	2016	2017	2018	2019	2020	2021	Average
Sichuan	0.769	0.754	0.755	0.756	0.769	0.722	0.747	0.761	0.730	0.730	0.738	0.748
Guizhou	0.170	0.158	0.205	0.285	0.317	0.357	0.357	0.335	0.347	0.337	0.313	0.289
Jiangsu	0.751	0.760	0.766	0.790	0.758	0.748	0.748	0.715	0.752	0.747	0.765	0.755
Anhui	0.443	0.430	0.463	0.477	0.467	0.487	0.475	0.481	0.554	0.557	0.563	0.491
Shanxi	0.343	0.321	0.290	0.230	0.271	0.264	0.249	0.237	0.247	0.258	0.227	0.267

**Fig 2:**
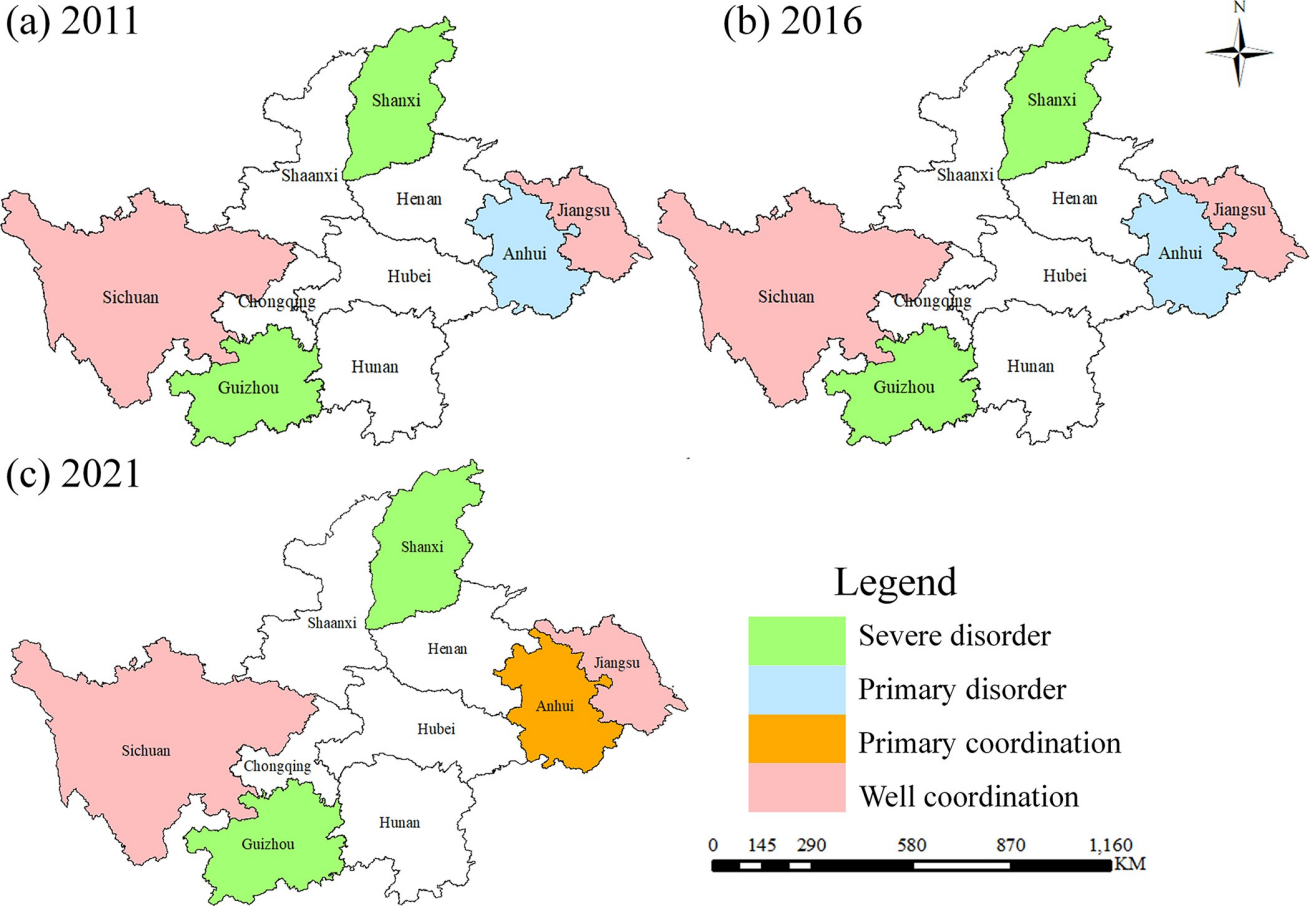
Spatial distribution of coupling coordination degree.

Due to the length of this study, we have selected only the years 2011, 2016 and 2021 to analyze the changes in the spatial distribution of coupling coordination. The spatial distribution is characterized by high east-west and low north-south (Fig 2). And in 2021, there is a pattern of coordinated development in Baijiu provinces along the Yangtze River basin. Sichuan and Jiangsu are well coordination, while Anhui is primary coordination. This pattern is in line with the reputation of the Yangtze River ecological brewing belt and famous Yangtze River liquor economic belt. From a provincial perspective, the evolution trend of Jiangsu and Sichuan is similar. Jiangsu has the highest mean coupling coordination degree (0.755), which can be attributed to differences in the STI foundation and development time sequence [53]. Although Guizhou and Shanxi are important Baijiu production areas, their STI foundation (Table 5) is poor and the comprehensive index score for the period 2011–2021 does not show significant improvement. This results in a mismatch between the development of the Baijiu industry and the STI foundation.

The base map outline was obtained by the Service Center of Standard Map (http://bzdt.ch.mnr.gov.cn/) and the permission number is GS (2016) 2923.

(2) Analysis of coupling coordination types

**Fig 3:**
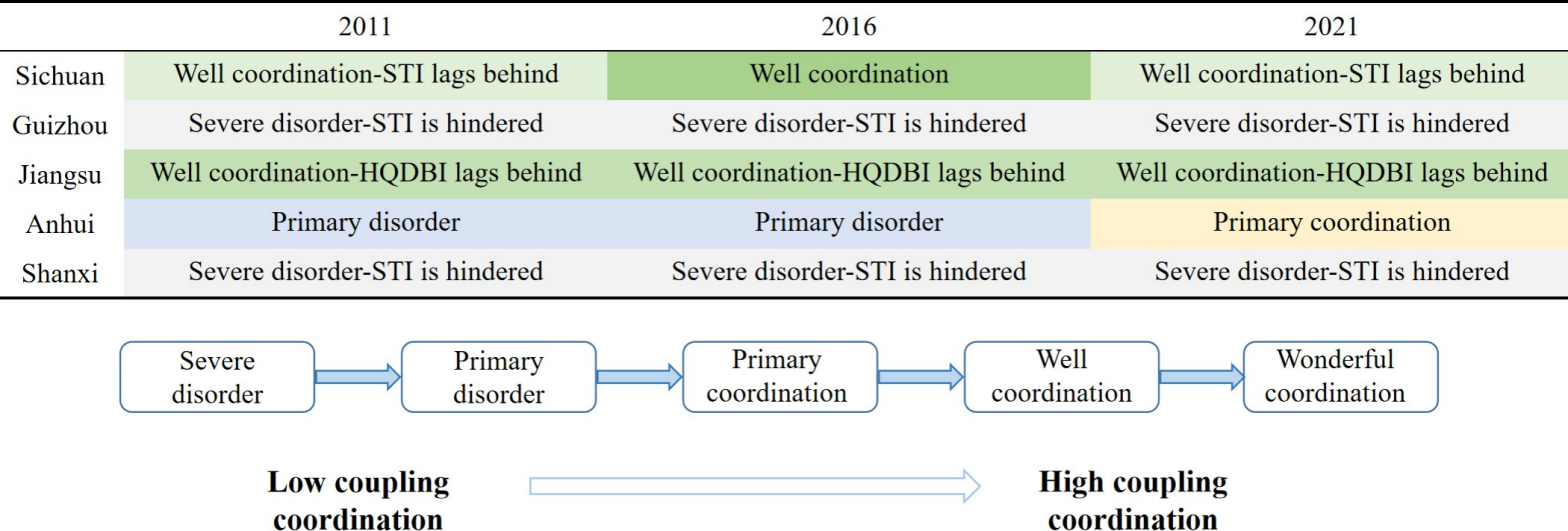
Coupling coordination subcategories.

The coupling coordination level of the five provinces shows small changes (Fig 3). Sichuan and Jiangsu exhibit more harmonious and stable levels of coordination. Jiangsu has well coordination, but HQDBI is lagging behind. Sichuan shows a fluctuating state of well coordination—STI lags behind and well coordination, which is related to the unique Baijiu brewing resources. The coupling coordination type between Guizhou and Shanxi is severe disorder—STI is hindered, and the coordination of STI and HQDBI is slow to develop. Anhui is the only province that has moved from disorder to coordination. Overall, there is still significant scope for improving the coordination of these provinces.

(3) Coordinating the influence of subsystems

First, the coordinating influence of STI on HQDBI subsystems.

**Fig 4:**
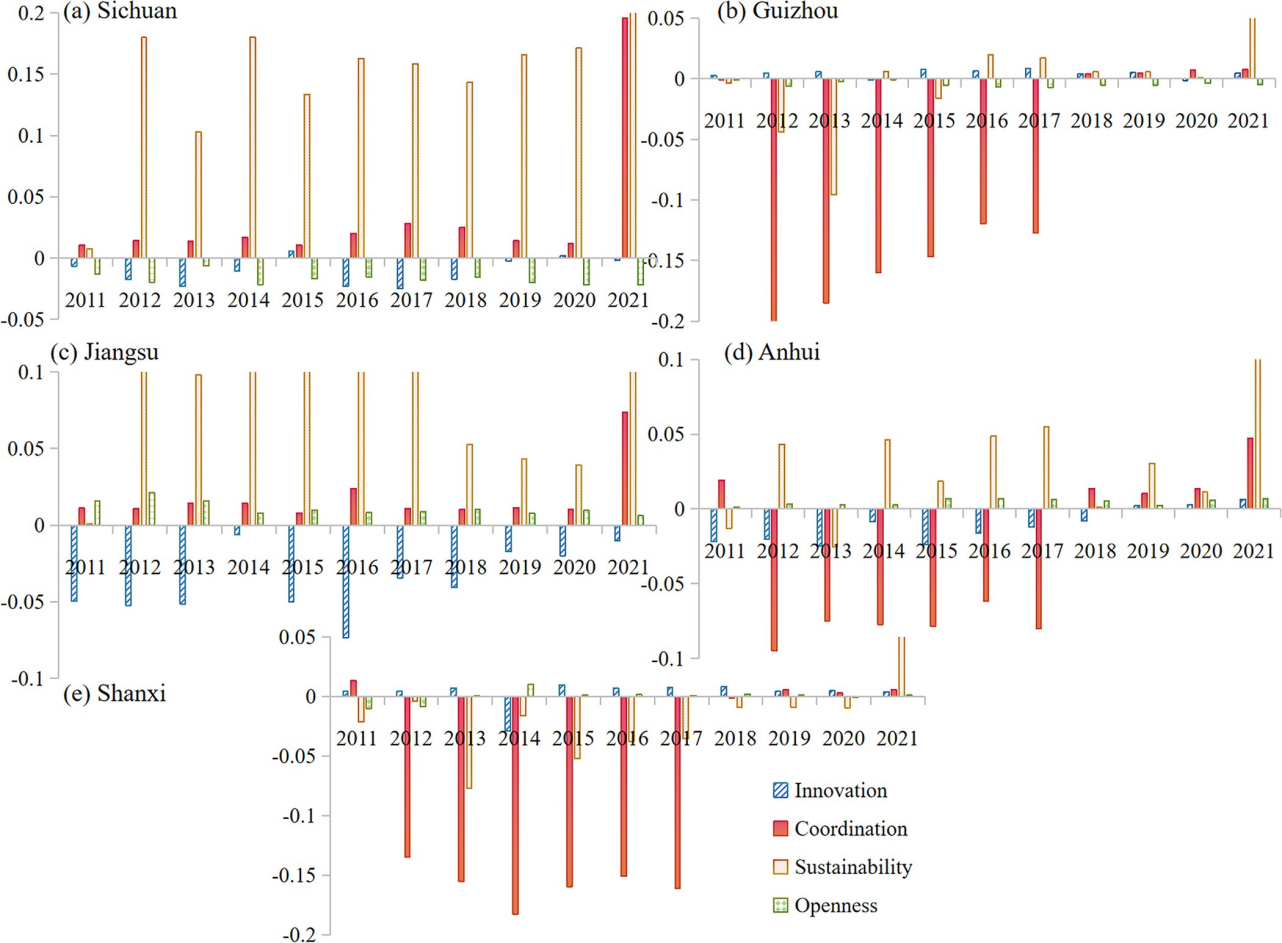
Coordinating influence of STI on HQDBI subsystems.

In the HQDBI subsystem, the coordinating influence of STI on innovation is mostly negative (Fig 4 and S3 File). This indicates that the coupling coordination degree of STI and innovation mainly plays a reverse constraint role. Coordination, sustainability and openness generally contribute positively to the degree of coupling coordination, and the strength of their contribution increases over time. The impact of STI on the coordination and sustainability subsystems varies across different provinces, with a stronger driving effect. Specifically, it has a positive facilitating effect on Sichuan and Jiangsu, while it mainly has a negative blocking effect on the comprehensive coordination degree in Guizhou, Anhui and Shanxi. However, it breaks through the zero value in 2018–2021 and shows a positive effect on facilitating coordination. It is evident that STI has a varied impact on coupling coordination at different stages. In addition, the direction of innovation is often opposite to coordination and sustainability. The differences in the degree of integrated coordination among provinces are primarily attributed to the checks and balances of these three subsystems. In summary, coordination and sustainability have a greater influence on the degree of coupling coordination and the effect is in the same direction (both negative). Changes in the degree of coordination in different regions depend largely on the synergy between the two main subsystems. It is evident that five provinces should commit to a coordinated and sustainable development path in the future. Enhancing coordination and sustainability has a positive impact on coordinating influence.

Second, the coordinating influence of HQDBI on STI subsystems.

**Fig 5:**
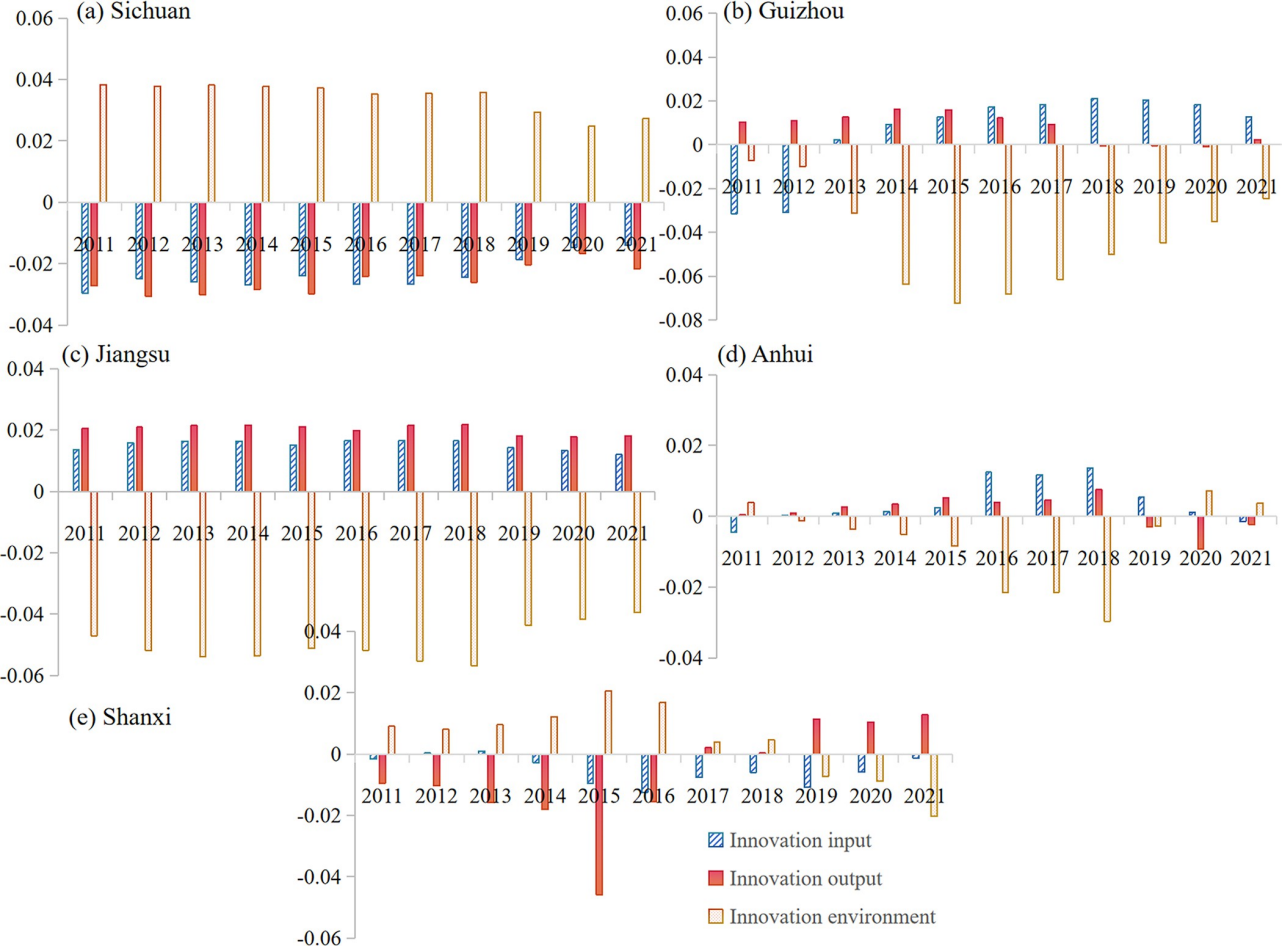
Coordinating influence of HQDBI on STI subsystems.

The influence of HQDBI on STI subsystems is coordinated by various factors. Among these, the innovation environment has a greater influence on the combined coupling coordination of provinces (Fig 5). The innovation environment subsystem is generally negative, except for some years in Sichuan and Shanxi. HQDBI mainly has a negative influence on the innovation environment. Regarding innovation inputs and outputs, the direction of influence is mostly consistent. However, innovation output is unstable, with the coordinated influence of the subsystem changing direction in many years. For example, Anhui has a positive coordinated impact on innovation output in 2011–2018, but it fell below zero in 2019, resulting in a significant negative impact.

### 4.3 Analysis of decoupling index and decoupling state

The decoupling states (Table 7) of STI and HQDBI are dominated by strong decoupling, strong negative decoupling and expansion negative decoupling. Strong decoupling occurs 16 times, indicating that HQDBI is no longer fully dependent on STI growth in most provinces. More specifically, the decoupling state shows differentiated characteristics for each province. Jiangsu has the smallest variation in the value of the decoupling index, ranging from -1.162 to 0.571. The decoupling index in Guizhou is significant variation and instability. The most frequent type of decoupling observed in Guizhou is expansion negative decoupling, with a positive growth rate for STI and HQDBI. This state indicates that the growth rate of HQDBI is lower than that of STI, indicating a positive interaction. The decoupling state of Sichuan is mainly strong decoupling and strong negative decoupling, and the decoupling index shows a fluctuating downward trend. In Shanxi, the decoupling states are recession decoupling and recession connection. This may be due to the strong influence of production areas, brand positioning and product reputation on the development of the Baijiu industry. Affected by market competition in advantageous production areas, Shanxi has intensified an unfavorable decoupling state in the short term.

**Table 7:** The decoupling index and decoupling state of STI and HQDBI.

Year	Decoupling index and decoupling state	Sichuan	Guizhou	Jiangsu	Anhui	Shanxi
2011	Index	-0.310	-1.724	-0.385	-3.490	0.892
	State	SN	SD	SD	SD	RC
2012	Index	-8.240	34.300	-0.264	-0.116	0.308
	State	SN	EN	SD	SD	WN
2013	Index	-2.282	-30.016	0.107	0.514	0.432
	State	SN	SN	WD	WD	WN
2014	Index	-0.048	6.297	-0.069	-0.284	-0.084
	State	SD	EN	SN	SN	SD
2015	Index	0.134	2.401	-0.242	3.789	-1.487
	State	WN	EN	SN	EN	SD
2016	Index	0.058	-1.234	-1.162	3.712	1.352
	State	WD	SD	SD	RD	RD
2017	Index	0.293	0.996	0.161	-0.551	-2.047
	State	WD	RC	WN	SD	SD
2018	Index	1.037	10.505	0.571	3.944	-3.644
	State	RC	EN	WD	EN	SN
2019	Index	-0.992	0.948	-0.295	-1.363	-0.176
	State	SD	RC	SN	SN	SD
2020	Index	-0.420	-4.157	0.227	1.801	0.783
	State	SD	SD	WD	EN	WN

The Baijiu industry is a traditional industry characterized by hand brewing and ancient brewing [42]. Balancing inheritance and technological innovation is a challenging problem that many Baijiu companies must confront. Baijiu industry needs to preserve the spirit of craftsmanship and traditional craft brewing. Despite changes in liquor demand and marketing, the current industry’s level of STI has not progressed significantly. Some preliminary research suggests that STI can enhance the optimization and upgrading of brewing ingredients, process control and food health [20]. Therefore, for Baijiu industry, it is preferable to maintain a weak decoupling of HQDBI and STI to stimulate economic vitality in the near future. This means that the development quality of HQDBI and STI is positive, and HQDBI develops faster than STI. This decoupling state is also more in line with the current situation of China’s Baijiu industry.

### 4.4 The transfer trend of coupling coordination degree

Spatio-temporal analysis can only simply depict the temporal trend and evolutionary differences of the coupling coordination degree. To gain a deeper understanding of its transfer law, the transfer trend was analyzed using the Markov chain. Based on the level of coupling coordination in 2011–2021, the Markov probability transfer matrix was constructed (Table 8).

**Table 8:** Markov probability transfer matrix.

	Low level	Medium-low level	Medium-high level	High level	Observed value
Low level	0.917	0.083	0	0	12
Medium-low level	0.143	0.714	0.143	0	14
Medium-high level	0	0.091	0.636	0.273	11
High level	0	0	0.308	0.692	13

The diagonal elements show the probability of the coupling coordination state remaining unchanged, reflecting the stability of the evolution of STI and HQDBI. Instead, the non-diagonal elements represent the probability of transfer between different states. The diagonal elements have a higher value than the off-diagonal elements (Table 8). This characteristic indicates that the coupling coordination degree is stable in maintaining the original state, with a probability of at least 0.636. The probability of moving up one level from low, medium-low and medium-high levels is 8.3%, 14.3%, and 27.3%. The coupling and coordination between the two systems is a dynamic process with twists and turns. The development dilemmas faced by different levels vary. The probabilities of shifting down one level for the low, medium-low and medium-high levels are 14.3%, 9.15% and 30.8%, respectively. This suggests that the five provinces are at a higher risk of a decline in the level of coupling coordination. Therefore, provinces should guard against a decline in the level of high coupling coordination. In addition, the probability of not being adjacent to the diagonal line is 0. This indicates that it is difficult to achieve a leapfrog development in the level of coupling coordination between consecutive years (e.g., progressing directly from a low level to a medium-high level).

### 4.5 The main factors that influence coupling coordination degree

Due to the large amount of data, this study shows only the average correlation of each indicator (Table 9). The correlation between the indicators and the coupling coordination degree in HQDBI system is greater than 0.5 [54], indicating a strong correlation between the selected indicators and the coupling coordination degree. The C3, C2, C6, C7, O1 and I2 indicators show the highest correlation among the five provinces, indicating that they are the primary influencing factors of HQDBI.

**Table 9:** The correlation between the indicators of HQDBI and the coupling coordination degree.

Province	I_1_	I_2_	I_3_	I_4_	C_1_	C_2_	C_3_	C_4_	C_5_	C_6_
Sichuan	0.829	0.882	0.693	0.693	0.881	0.882	0.895	0.878	0.890	0.884
Guizhou	0.649	0.655	0.747	0.676	0.844	0.860	0.737	0.718	0.802	0.859
Jiangsu	0.805	0.897	0.890	0.844	0.942	0.886	0.929	0.938	0.928	0.946
Anhui	0.818	0.673	0.710	0.577	0.828	0.829	0.835	0.836	0.837	0.837
Shanxi	0.887	0.910	0.816	0.755	0.886	0.901	0.787	0.776	0.777	0.897
Province	C_7_	S_1_	S_2_	S_3_	S_4_	S_5_	S_6_	O_1_	O_2_	O_3_
Sichuan	0.736	0.848	0.893	0.808	0.882	0.877	0.869	0.857	0.689	0.718
Guizhou	0.801	0.762	0.818	0.672	0.776	0.842	0.754	0.682	0.667	0.777
Jiangsu	0.946	0.929	0.930	0.926	0.839	0.847	0.928	0.924	0.877	0.818
Anhui	0.841	0.779	0.781	0.778	0.821	0.829	0.736	0.848	0.823	0.648
Shanxi	0.900	0.901	0.899	0.753	0.740	0.761	0.819	0.898	0.783	0.832

**Fig 6:**
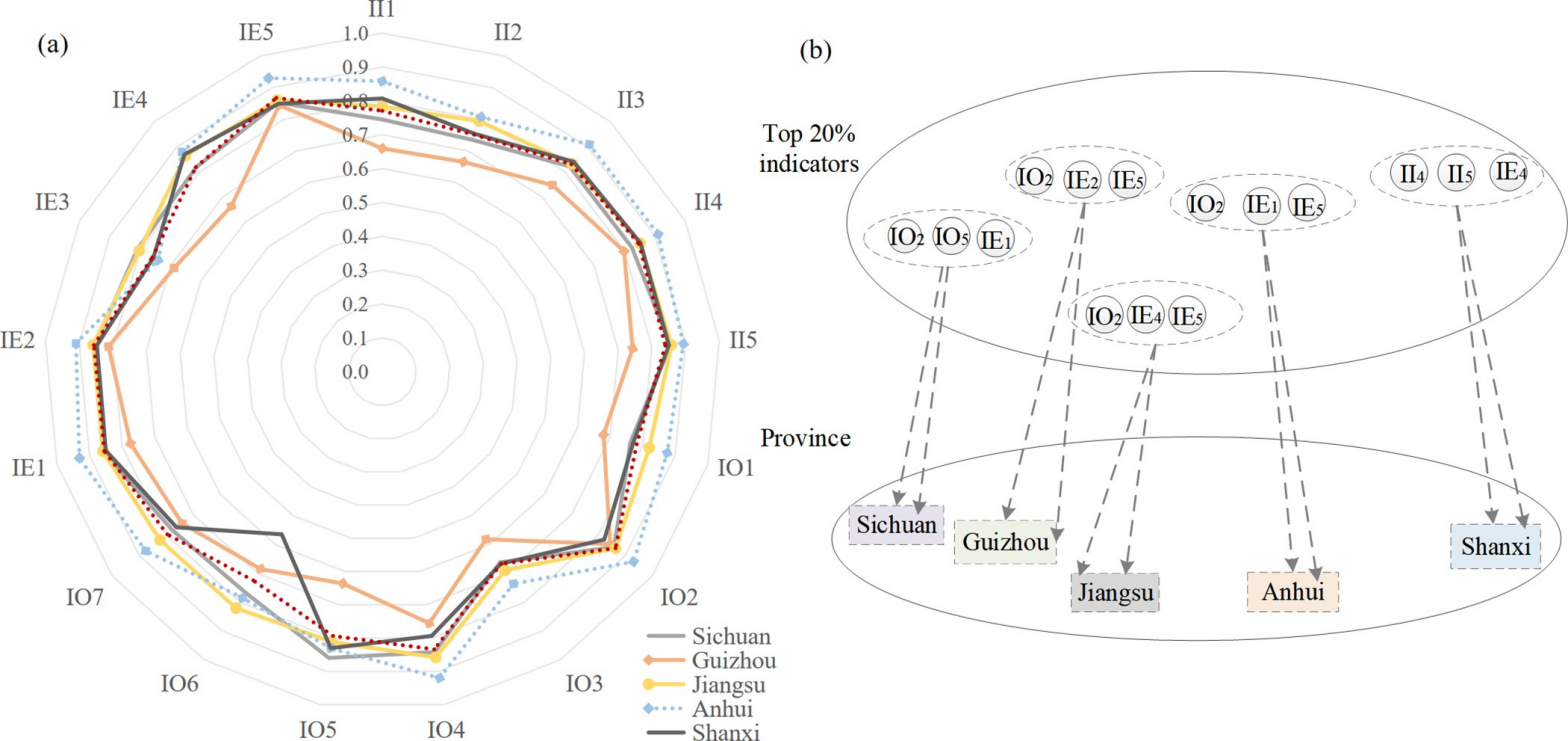
Grey correlation results of STI system and coupling coordination. (a) Radar chart of correlation; (b) Top 20% of indicators.

For STI system, key factors are selected based on indicators that rank in the top 20% of correlation among provinces [72]. Fig 6 shows that the key influencing factors are IO2, IO5, IE1, IE2, IE5, IE4, II4 and II5.

## 5 Discussion

### 5.1 Based on the coupling perspective

During the comprehensive revitalization of Baijiu development, liquor-producing provinces face the challenge of balancing industrial development with STI to achieve the coordinated development of STI and HQDBI. This study used the coupling coordination model and the coordinating influence model to measure the influence relationship between STI and HQDBI in several provinces in China. The results indicate that there is a severe disorder in Guizhou and Shanxi. The hindrance of STI appears to be the main cause. Apparently, the development foundation of Baijiu industry, the level of STI and the economic status in these regions differ significantly, resulting in varying degrees of coordination [21]. In addition, this study also concludes that there is little difference in the spatial distribution of coupling coordination in the same region. This means that the change in the coupling coordination degree is a gradual process. And there may be similarities and correlations in their coupling coordination mechanisms [24]. The results of the Markov chain (Table 8) also support this conclusion.

For Baijiu industry, there is a ’hypothesis of STI promoting economic growth of Baijiu industry’ [48]. However, some scholars disagree with this view. They argue that the development of STI may increase industrial production and energy consumption [37]. It may even lead to industrial overdevelopment and affect the long-term stability of the economy [73]. The results of this study are very consistent with the former view. In 2021, the spatial distribution results are consistent with the pattern of coordination development of Baijiu provinces along the Yangtze River Basin (Fig 2). That is, Chinese Baijiu has obvious geographical landmarks, which confirms the findings of Barham (2003) [74].

Second, unlike existing studies that follow the trend of disorder to coordination [21], the coordination of Baijiu industry and STI coupling between Guizhou and Shanxi is still in the disorder stage from 2011 to 2021. This may be attributed to the ’restriction of three public expenditures’ (i.e., limiting the consumption of high-priced Baijiu by public funds, etc.). This indicates that the level of coupling coordination of Baijiu industry still needs to be improved. However, the ’Baijiu production line restriction’ has been lifted [20] (since 2020, it has been removed from the restricted category). We believe that the coupling degree between STI and HQDBI will improve in the future. In addition, five provinces have not achieved high-quality coordination between STI and HQDBI. This contrasts with previous research conclusions that found Sichuan and Jiangsu achieved wonderful coordination during the 12th Five-Year Plan period [70]. The discrepancy may be due to differences in the sample base period, rendering the findings incomparable in the time dimension.

Finally, the coupling coordination of STI and HQDBI is the result of multiple factors [21]. Different influencing factors can be analyzed from the subsystems of STI and HQDBI, respectively. It is worth noting that the main driving (or blocking) forces in the Baijiu industry system are coordination and sustainability (Fig 4). Factors related to the coordination and sustainability of Baijiu industry, such as C2, C3, C6 and C7, have superior performance in these regions.

### 5.2 Based on the decoupling perspective

The decoupling relationship between STI and HQDBI is constantly changing, and there exists a dynamic decoupling of relative and absolute decoupling [75]. STI and HQDBI exist in multiple types of decoupled states [35], including strong decoupling, expansion negative decoupling, weak decoupling and strong negative decoupling. However, this study did not conclude a continuous decoupling process from weak decoupling to strong decoupling, which differs from previous research [61]. The Baijiu industry (a traditional industry) differs from high-tech industries [9]. Currently, the level of STI is still low [70]. The intelligent control of brewing raw materials and processes is still being optimized [20]. A single decoupled state is not conducive to the transformation and development of the Baijiu industry in the near future.

The innovation of brewing technology and the transformation of STI achievements in Baijiu industry are the key points. For example, regions with a strong foundation of STI and Baijiu industry, such as Sichuan and Jiangsu, have a relatively high comprehensive index. On the positive side, the increase in the output of Baijiu industry is beneficial to economic growth and technological progress. The progress of STI has led to a virtuous cycle of HQDBI. The decoupling results also confirm this conclusion. The decoupling state is mainly strong decoupling. This means that HQDBI is not fully dependent on the growth of STI in most provinces. It also implies that large STI inputs do not necessarily lead to higher Baijiu outputs and revenues, which can only be realized if STI resources are allocated appropriately [76]. These findings may be related to some national strategies, such as industrial structure adjustment and optimization [6]. They may also be associated with well-known Baijiu techniques, such as hand brewing [42].

However, it is worth noting that regional imbalances and inconsistencies continue to be a significant issue. Specifically, Guizhou and Shanxi are not yet ideal decoupling states. For the entire study region, expanding negative decoupling and strong negative decoupling still account for a large proportion, and the growth rate of HQDBI will slow down to below the growth rate of STI. The current decoupling state is not beneficial for the development of Baijiu industry in these two provinces.

## 6 Conclusion

The main conclusions are as follows:

1. In 2011–2021, the level of STI and HQDBI shows an overall upward trend in five provinces. However, there are significant differences between regions. Sichuan and Guizhou have a higher comprehensive index of STI and HQDBI compared to Jiangsu, Anhui and Shanxi.
2. The change in coupling coordination degree in five provinces is relatively small and has not yet demonstrated a wonderful coordination pattern. The spatial distribution presents the characteristics of high east-west and low north-south. In 2021, a pattern of coordinated development in Baijiu provinces along the Yangtze River basin has emerged. The coordinated influence of the subsystem is greatly influenced by the coordination, sustainability and innovation environment.
3. The decoupling state presents differentiated characteristics, including strong decoupling, strong negative decoupling and expansion negative decoupling. The regional imbalance and uneven characteristics of HQDBI and STI still require attention from relevant departments.
4. The coordination process between STI and HQDBI is complex and dynamic, with an unstable phenomenon that makes achieving leapfrog development difficult. Each level of coordination has the risk of downward transfer.

However, this study also has certain limitations. Although the coordination between STI and HQDBI was explored from the perspective of coupling and decoupling, their internal operating mechanisms were not analyzed in depth. Future research should analyze the mechanisms of STI and HQDBI more thoroughly.

## Supporting information

S1 File Calculation results of entropy-improved CRITIC. (XLSX)

S2 File The results of coupling coordination and Tapio decoupling model. (XLSX)

S3 File The result of coordinating influence model. (XLSX)
